# Gas Plasma-Induced Oxidative Transformation of Glucose

**DOI:** 10.3390/biomedicines13112833

**Published:** 2025-11-20

**Authors:** Mohsen Ahmadi, Kai Masur, Sander Bekeschus, Kristian Wende

**Affiliations:** 1 ZIK Plasmatis, Leibniz Institute for Plasma Science and Technology (INP), Felix Hausdorff-Str. 2, 17489 Greifswald, Germany; 2 Department of Dermatology, Venerology, and Allergology, Rostock University Medical Center, Strempelstr. 13, 18057 Rostock, Germany

**Keywords:** kINPen, reactive oxygen species, oxidation, ROS, glucose, glucose oxidation products (GOPs)

## Abstract

**Background**: Glucose, a central carbohydrate in higher organisms’ metabolism, can undergo extensive oxidative modification under conditions of excessive inflammation or elevated reactive oxygen and nitrogen species (RONS). Such modifications yield glucose oxidation products (GOPs) with potential biological relevance and toxicity. This study aimed to systematically characterize GOP formation under defined oxidative conditions generated by gas plasma treatment. **Methods**: *D*-glucose solutions were prepared at 0.25 mM (hypoglycemic/diabetic range), 2.5 mM (sub-physiological), and 25 mM (peritoneal dialysis fluid). Samples were exposed for up to 20 min to the atmospheric-pressure argon plasma jet kINPen, which produces a wide spectrum of RONS. Treatment time-dependent glucose oxidation was assessed by high-resolution mass spectrometry (HRMS) and tandem mass spectrometry (MS/MS) to identify the oxidation products. **Results**: Gas plasma exposure generated various oxidation products and their abundance profiles depended on initial glucose concentration and treatment duration. Identified products included 2-keto-*D*-glucose, 3-deoxyglucosone (3DG), 3,4-dideoxyglucosone-3-ene (3,4DGE), furaldehyde, methylglyoxal, and acetaldehyde. HRMS/MS analysis confirmed diagnostic fragment ions for each GOP and revealed distinct formation across the model scenarios. **Conclusions**: Cold gas plasma induces a spectrum of glucose oxidation products under biomedically relevant glucose levels. The identified GOPs, many of which have known cytotoxic or signaling properties, provide mechanistic insight into glucose oxidation in inflamed or oxidative microenvironments. These findings support the utility of plasma-based oxidative models for studying GOP-associated biological effects and potential pathophysiological consequences.

## 1. Introduction

**Figure 1:**
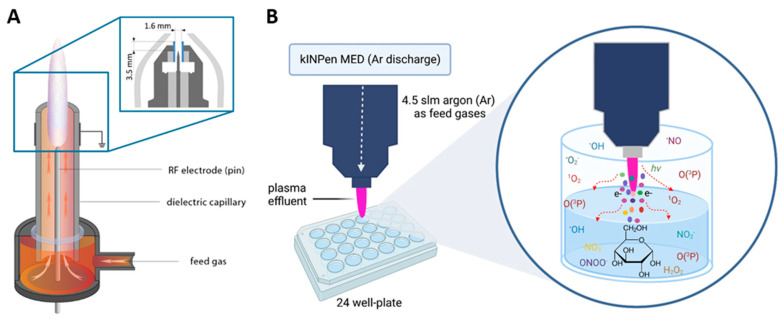
(**A**) The kINPen schematics [9] (inset: the kINPen head geometry with a gas curtain device (light gray), electrodes (dark gray), and a dielectric capillary (blue)). Adapted in part from Ref. [9] under the Creative Commons Attribution 3.0 license, (**B**) Schematic illustration for the plasma treatment in this work. slm = standard per liter, Ar = argon. Plasma nozzle distance from the liquid surface: 9 mm.

In recent years, plasma technology has gained significant attention for its potential applications in various fields, such as chronic wound healing [1], cancer therapy [2], prodrug activation [3], lipid [4] and peptide [5] oxidation, immunogenic protein modification [6], and catalytic processes [7]. Cold gas plasma, a partially ionized gas, is known to generate a diverse range of reactive oxygen and nitrogen species (RONS) capable of interacting with biological systems [8]. Various short- and long-lived RONS, such as singlet oxygen (^1^O_2_), hydroxyl radical (^•^OH), atomic oxygen (O(^3^P)), superoxide anion radicals (^•^O_2_^−^), ozone (O_3_), nitric oxides (N_x_O_y_), and hydrogen peroxide (H_2_O_2_) alongside free electrons, (vacuum-) UV radiations and electromagnetic fields, and neutral particles can be generated by plasma jets such as the kINPen [9] (Figure 1A). An intriguing area of investigation concerns the impact of plasma-generated RONS on carbohydrates, with *D*-glucose serving as a representative model compound. Glucose, a key carbohydrate and primary energy source for living organisms, can undergo various chemical reactions when exposed to plasma-induced RONS. The chemical impact of gas plasma on carbohydrates was previously reported, including *D*-glucose oxidation upon argon and argon-oxygen kINPen plasma jet exposure [10], and *D*-ribose, *D*-glucose, and *D*-sucrose generation upon air dielectric-barrier discharge plasma treatment [11].

The exposure of *D*-glucose to plasmas like the kINPen leads to the formation of numerous GOPs. Some of these products are reported as toxic, like 3-deoxyglucosone (3DG) and 3,4-dideoxyglucosone-3-ene (3,4DGE) [12,13,14], and have the potential to exert detrimental effects on cellular systems. Plasma-induced reactive species such as ^•^OH and O(^3^P) possess strong oxidizing properties [15,16]. Considering this existing knowledge on the mechanism of reactive species-based oxidative modifications, we intended to shed more light on the potential formation of toxic GOPs. In this study, we explored plasma–glucose interactions under different glucose concentrations (0.25, 2.5, and 25 mM) [17], representing (hypoglycemic) diabetic, (sub-) physiological, and peritoneal dialysis fluid conditions, respectively, when exposed to RONS. This approach aimed to assess whether such oxidative processes occur to a similar extent across biologically and technically relevant environments. To investigate this, glucose solutions were oxidized by plasma-generated reactive species using the medical device kINPen MED, and the resulting products were analyzed by high-resolution mass spectrometry (HRMS) and tandem mass spectrometry (MS/MS) (Figure 1B).

## 2. Materials and Methods

### 2.1. Sample Preparation

A 50 mM stock aqueous solution of *D*(+)-Glucose (Sigma-Aldrich Chemie GmbH, Taufkirchen, Germany) was prepared. The glucose solution was diluted in water to final concentrations of 0.25, 2.5, and 25 mM in a 24-well plate (Sarstedt AG & Co. KG, Nümbrecht, Germany) before each experiment. During the setup, gas flow-induced evaporation was compensated for by adding a predefined volume of water to maintain constant concentrations. The temperature of the treated liquid did not increase. Samples treated with the kINPen Med for 5–20 min were collected, divided into aliquots, and diluted 1:1 (*v*/*v*) with MeOH:H_2_O containing 0.05% formic acid (FA) to yield a final concentration of 125 μM before MS analysis. All samples were incubated at ambient temperature for 2 h (corresponding to the overall sample preparation time) before mass spectrometric analysis to minimize secondary reactions or degradation processes that could affect the composition of the oxidation products.

### 2.2. Gas Plasma Exposure

All experiments were performed at ambient temperature using a kINPen MED with argon (Ar) as feed gas (kINPen; neoplas MED GmbH, Greifswald, Germany). The kINPen device comprises a grounded ring electrode that encloses a ceramic capillary with a diameter of 1.6 mm. Inside the capillary is a powered central rod electrode operating at a frequency of 1.1 MHz and a voltage ranging from 2 to 6 kVpp. The gas flow was maintained at a constant rate through the capillary, allowing efficient interaction between the plasma-generated reactive species and the liquid phase, including dissolved components [18]. The gas flow rate was set at 4.5 standard liters per minute (slm) of pure Ar (99.999% purity; Air Liquide S.A., Paris, France) that served as feed gas. The gas plasma is generated at the tip of the central electrode and expands into the ambient air. A 600 μL volume of aqueous glucose solution was placed in a 24-well plate and treated with a plasma jet nozzle at a liquid distance of 9 mm for all plasma treatments.

### 2.3. Mass Spectrometry (MS)

To elucidate the GOPs, high-resolution mass spectrometry (HRMS) and tandem mass (MS/MS) were performed using a TripleTOF 5600 mass spectrometer (AB Sciex, Darmstadt, Germany). All prepared samples at a final concentration of 125 μM in MeOH:H_2_O (1:1, *v*/*v*) containing 0.05% FA were directly infused into the Turbo Ion Source at a flow rate of 7 μL/min using negative electrospray ionization (ESI). Survey spectra were acquired over an *m*/*z* range of 30–1000, and selected peaks were fragmented by collision-induced dissociation (CID). The mass spectrometer was calibrated with a Sciex tuning solution (ESI Negative Calibration Solution). All measurements were conducted under identical conditions: capillary temperature 250 °C, curtain gas (N_2_) 35 psi, ion source gas 2 (N_2_) 35 psi, and ion spray voltage 3.8 kV. The MS/MS spectra of oxidation products were acquired in product ion mode (collision energies: 10–70 eV, declustering potential: 80 V). Calibration was performed prior to measurements using external calibration curves generated from standard samples. Peak areas for all spectra were calculated, and duplicates were presented with their standard deviations. External calibration curves were then used for quantification.

### 2.4. Data Analysis and Visualization

Data acquisition, mass signal integration, and calculation were performed by using Analyst TF 1.71 software (AB Sciex), Peakview software (version 1.1.1.2, AB Sciex), and Prism (version 9.2.0; GraphPad Software, Boston, MA, USA). Glucose oxidation and mass spectrometry experiments were performed twice, each with technical duplicates unless otherwise indicated.

## 3. Results and Discussion

In this study, glucose oxidation was conducted using argon (Ar)-driven kINPen plasma jet (kINPen MED). Previous studies have demonstrated that parameters such as gas composition, flow rate, nozzle-to-surface distance, and exposure duration influence plasma-induced oxidation processes [3,5,19]. These parameters determine the concentration and the chemical nature of the reactive species produced by the plasma [20]. We used defined *D*-glucose concentrations to model relevant physiological contexts: 0.25 mM (hypoglycemic), 2.5 mM (sub-physiological), and 25 mM (peritoneal dialysis fluids, providing a basis to assess plasma-induced oxidative effects on glucose. These concentrations reflect glucose levels in various real-world settings, enabling an investigation into how plasma-induced oxidative processes impact glucose. This approach provided a comprehensive assessment of the glucose oxidation products (GOPs) formed by plasma-induced ROS across different glucose levels, offering valuable insights into potential effects on biological systems and applications.

**Figure 2:**
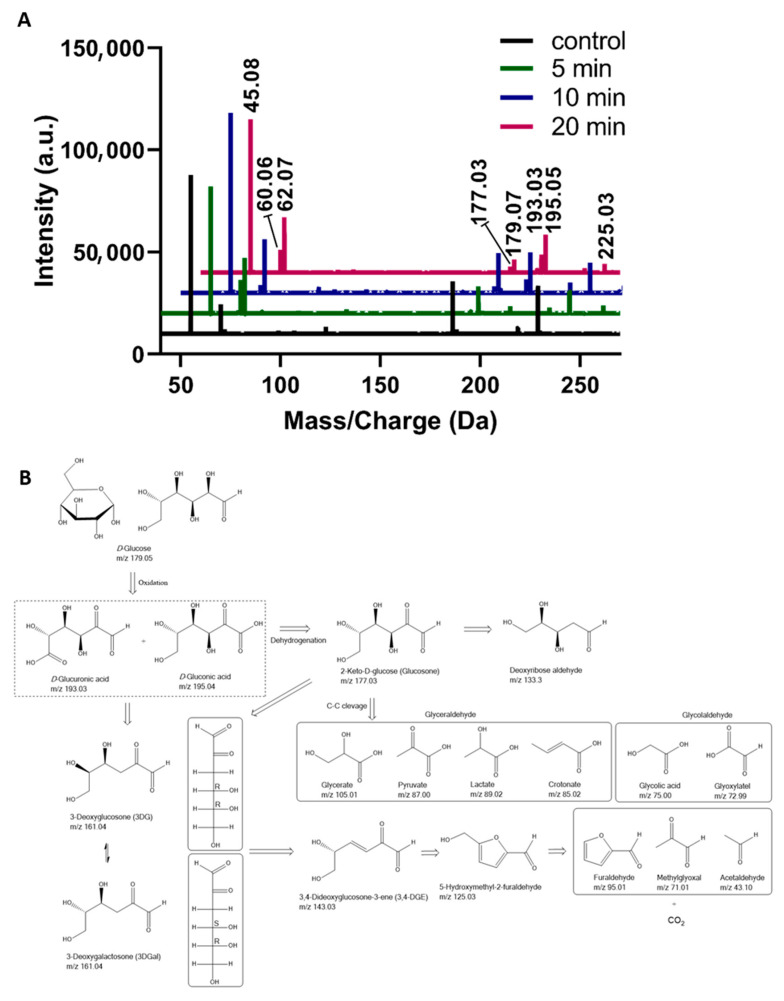
(**A**) HRMS spectra of *D*-glucose solution (2.5 mM) after plasma treatment for 10 min. (**B**) Plausible mechanistic interpretation of glucose oxidation products [26,29,30]. Inserted box: 3DGal is the C4 epimer of 3-DG.

To infer on the GOPs, HRMS analysis of *D*-glucose solutions after plasma treatment was conducted. The MS spectra of 2.5 mM *D*-glucose solutions after plasma treatment for 5, 10, and 20 min vs. untreated samples are shown in Figure 2A. The *D*-glucose MS signal was observed at *m*/*z* 179.05 [C_6_H_12_O_6_ − H]^−^. Another MS signal observed at *m*/*z* 225.05 corresponds to [C_6_H_12_O_6_ + HCOO]^−^, with the monoisotopic mass of formate being 44.9982. Plasma treatment led to a decomposition of glucose, reflected by a reduction in glucose signals across all tested concentrations (Supporting Information, Figure S1). The strongest effect was observed at the lowest concentration (0.25 mM), where the limited amount of substrate was rapidly consumed by plasma-generated RONS. As a result, the formed GOPs were further exposed to oxidative stress during the treatment, leading to their subsequent degradation under the same plasma conditions. Across all treatment durations and glucose concentrations, *D*-gluconic acid (*m*/*z* 195.04) and *D*-glucuronic acid/*L*-guluronic acid (*m*/*z* 193.03) represented the main detectable GOPs, confirming their central role as primary intermediates in plasma-induced glucose oxidation. A plausible mechanistic explanation for the plasma-induced oxidation of glucose is a sequence of oxidative steps involving oxygen addition, dehydrogenation, and subsequent C-C bond cleavage, resulting in the release of CO_2_ (Figure 2B). This aligns with degradation pathways described in other oxidation-driven transformations of free carbohydrates [21,22,23]. The primary oxidation of glucose represents intermediates, which can further undergo dehydrogenation to form 2-keto-*D*-glucose (glucosone). With prolonged treatment, the increase in the number of MS signals indicated the formation of multiple low-abundant GOPs. The oxidation primarily affected the anomeric carbon and adjacent hydroxyl (−OH) groups of glucose and its oxidation products, promoting ring opening and the subsequent generation of aldehyde (−CHO) and carboxylic acid (−COOH) derivatives [24]. Further oxidative cleavage of these intermediates, such as gluconic and glucuronic acids, likely led to decarboxylation and CO_2_ loss. The signal at *m*/*z* 177.03 was prominent, corresponding oxidation product of *D*-gluconic acid (*m*/*z* 195.04): 2-keto-*D*-glucose ([C_6_H_10_O_6_ − H]^−^). Although the signal at *m*/*z* 177.03 could also indicate the presence of isobaric glucono-1,4- or 1,5-lactones, multiple observations support its assignment to 2-keto-*D*-glucose. First, the MS/MS spectrum matched that of a commercial 2-keto-*D*-glucose standard (see Table 1; Figures S8 and S9). Second, under the acidic conditions generated during plasma treatment (pH~3.4; Figure S7), glucono-lactones are expected to hydrolyze rapidly to gluconic acid [25], consistent with the abundant gluconate signal (*m*/*z* 195.05). The presence of dimeric and trimeric adducts (*m*/*z* 355.11, 533.17) with coherent MS/MS further supports 2-keto-*D*-glucose formation. Further oxidation yields α-dicarbonyl compounds like 3-deoxyglucosone (3DG) or 3-deoxygalactosone (3DGal) (161.04 *m*/*z*) in different pathways. 3DGal is less stable than 3-DG, influencing formation and degradation pathways. Hellwig et al. [26] reported the possible formation of 3-DGal from 3-DG through the intermediate molecule 3,4-dideoxyglucosone-3-ene. However, 3DG formed by glucose degradation via enolization/dehydration processes is a key intermediate in glucose degradation [27]. Both 2-Keto-*D*-glucose (177.03 *m*/*z*) and 3,4-dideoxyglucosone-3-ene (3,4DGE) (143.03 *m*/*z*) could further oxidize into smaller aldehyde derivatives such as methylglyoxal (71.01 *m*/*z*) via cyclization and furaldehyde (95.01 *m*/*z*) via carbon-carbon bond cleavage. In addition, some minor interesting signals emerged, including *m*/*z* 149.00 (bitartrate, C_4_H_5_O_6_^−^) and *m*/*z* 161.00 (C_5_H_5_O_6_^−^, possibly hydroxyoxoglutarate-related compounds), which appeared predominantly after prolonged exposure (≥10 min). These findings highlight the dynamic nature of glucose oxidation, particularly at low substrate concentrations, where early oxidation products (*m*/*z* 193.03 and 195.04; *D*-gluconic and *D*-glucuronic acid) decrease with treatment time, indicating their further conversion (degradation) under continued oxidative stress. Altogether, these transformations reflect an oxidative degradation of glucose, where RONS-mediated electron abstraction and C–C scission drive the conversion from glucose to short-chain hydroxy/sugar acids and aldehydes [28]. The major oxidation modifications of *D*-glucose detected after plasma exposure are listed in Table 1. Representative mass spectra of untreated and plasma-treated 0.25 mM *D*-glucose solutions are provided in the Supporting Information (Figures S2–S6).

**Figure 3:**
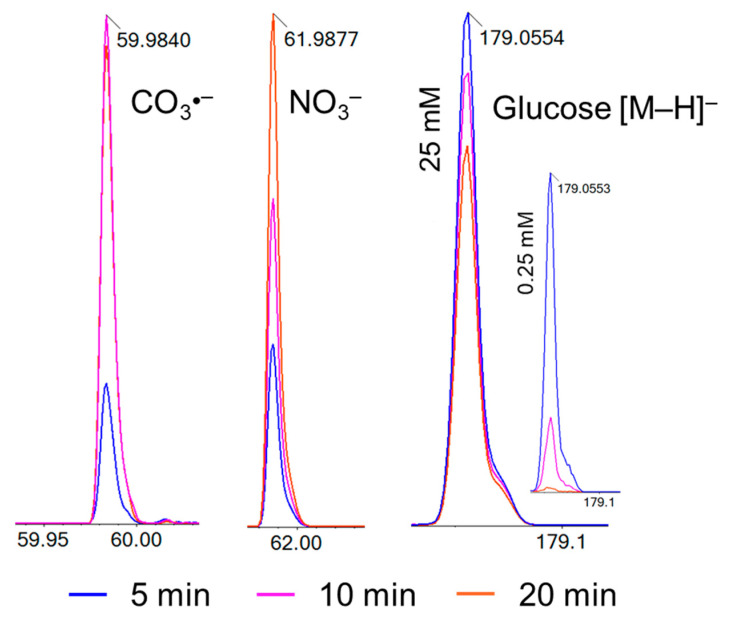
Production of CO_3_^•−^ (**left**), NO_3_^−^ (**middle**), and glucose decomposition (**right**) during plasma treatment from 5 to 20 min, TOF-MS signal intensities. Glucose decomposition efficacy inversely correlates with initial concentration (25 mM large graph; 0.25 mM insert).

After plasma treatment, significant quantities of carbonate radicals (CO_3_^•−^; *m*/*z* 59.98) and nitrate (NO_3_^−^; *m*/*z* 61.99), were detected (Figure 3), while glucose molecules (*m*/*z* 179.05) were decomposed (Figure 2A and Figure 3). During plasma exposure, the pH of the treated aqueous solutions decreased substantially, from 5.8 in the untreated glucose solution to 3.4 after 20 min of plasma treatment (Supporting Information, Figure S7), consistent with our previously reported plasma-induced acidification in aqueous systems [10]. This acidification primarily arises from the dissolution of plasma-generated NO and NO_2,_ forming nitric and nitrous acids (HNO_3_ and HNO_2_) [31], and secondarily from organic acid formation (e.g., formic, glycolic, and glyoxylic acids) during glucose oxidation [32]. As the treatment time increases, the peak area of the MS signal for CO_3_^●−^ and NO_3_^−^ increases (Figure 3, Table 2). Peroxynitrite is a strong oxidant that contributes to glucose decomposition. At the same time, oxidation-sensitive nitrite (NO_2_^−^, *m*/*z* 45.99), which is frequently detected in (buffered) liquids treated by argon plasmas like the kINPen, was observed in traces only reflecting the strongly oxidative environment created. Bicarbonate (HCO_3_^−^; *m*/*z* 61.06) present in the solution can react with hydroxyl radicals (^•^OH) to generate CO_3_^•−^, which also contributes to glucose decomposition [33]. Since carbonate radicals have a millisecond lifetime, the radical ions detected in mass spectrometry were generated in the electrospray ion source and represent carbonate ions. The non-monotonous change in their concentration with treatment time can be explained by (bi-) carbonate formation during glucose decomposition (decarboxylation of α-oxo carbonic acids), and their further decay. Carbonate radicals formed by the reaction between carbonate ions and hydroxyl radicals during plasma treatment may contribute to the glucose decomposition. Since they have a very low pKa (<0), their reactivity is independent of pH value, while the reactivity of target molecules, e.g., carboxylic acids, changes [34].

The key GOPs formed by plasma-induced RONS are summarized in Table 1. Tandem MS analysis, using commercially available standards, confirmed the formation of the oxidation products. The MS/MS spectrum of the ion at *m*/*z* 177.03 showed fragment ions at 159.02, 129.01, 117.01, 99.00, 87.00, 71.01, and 59.01, matching the fragmentation pattern of 2-keto-*D*-glucose analyzed under identical conditions. (Supporting Information, Figures S8 and S9). *D*-Gluconic acid with an *m*/*z* of 195.04, the MS/MS fragmentation produces ions at 177.03, 129.01, 99.00, 87.00, 85.00, 75.00, 59.01, and 57.00. These fragments confirmed the structure and identity of gluconic acid and 2-keto-*D*-glucose. The hydroxyl group at the C2 position of *D*-gluconic acid undergoes oxidation, resulting in the formation of a carbonyl group at that position and the subsequent formation of 2-keto-*D*-glucose. In principle, *D*-glucuronic acid and 2-deoxy-*D*-glucose (2DG) are derivatives of glucose but differ in structure and function. *D*-Glucuronic acid produced a characteristic MS/MS spectrum from the precursor ion at *m*/*z* 193.03, yielding fragment ions at 165.03, 147.02, 131.03, 113.02, 103.00, 89.02, 85.02, 73.00, 71.01, 59.01, and 45.00. This compound arises from oxidation at C6, where the terminal hydroxyl group is converted into a carboxylic acid. Arabinonate (C_5_H_9_O_6_^−^, *m*/*z* 165.03) was also identified as one of the glucose oxidation products, indicating oxidative cleavage and partial chain shortening of the parent structure. 2DG is a deoxy sugar in which the hydroxyl group at C2 is replaced by hydrogen, resulting in a lower molecular mass (*m*/*z* 163.06). In the present analysis, no signal corresponding to 2DG was observed, indicating that under the applied oxidative conditions, deoxygenation of glucose at C2 did not occur. The signal observed at *m*/*z* 163.02 corresponds to aldehydo-arabinuronate, the open-chain (aldehyde) form of arabinuronic acid. This compound can form through oxidative C-C bond cleavage of glucose or gluconic acid, involving the loss of one carbon atom and concurrent oxidation of the terminal hydroxyl group. Interestingly, the MS signal at *m*/*z* 71.01 was detected as a fragment ion for the parent ions in the MS/MS spectra of the signals at *m*/*z* 193.03, 177.03, 163.01, 161.06, and 143.03. The significance of *m*/*z* 71.01 lies in its identification as a characteristic ion of methylglyoxal [C_3_H_4_O_2_ − H]^−^, a reactive compound that can be formed through the oxidative degradation pathways of carbohydrates, which contribute to oxidative stress and cellular damage, especially in diabetic complications [35,36,37]. Other signals observed in the HRMS spectra are also associated with reactive functional groups present in glucose oxidation products, suggesting their potential toxic effects on biological systems. For example, previous studies have demonstrated that the 3DG and 3,4DGE present in heat-treated sterile peritoneal dialysis fluids exhibit cytotoxicity on cultured adipose progenitor cells, as evidenced by the inhibition of cell growth measured through total protein quantification [13,38,39,40].

The relative abundance of oxidation products was analyzed after plasma treatment, and the results were expressed as percentages based on the peak area of the glucose molecule ion (*m*/*z* 179.05). The degradation of *D*-glucose increases progressively with longer treatment times and lower glucose concentrations (Figure 3). It can be attributed to the continuous interaction of reactive species with the glucose molecules present in the solution. The difference in oxidation efficiency between the glucose solutions is rooted in the concentration of glucose molecules and their interaction with plasma-induced reactive species. In lower concentrations (0.25 mM and 2.5 mM), a limited number of glucose molecules are present, increasing the likelihood of interacting with abundant RONS, resulting in a substantial oxidation of glucose and its primary oxidation products. In contrast, at 25 mM, the higher number of glucose molecules consumes more RONS, reducing the oxidative impact per molecule.

**Figure 4:**
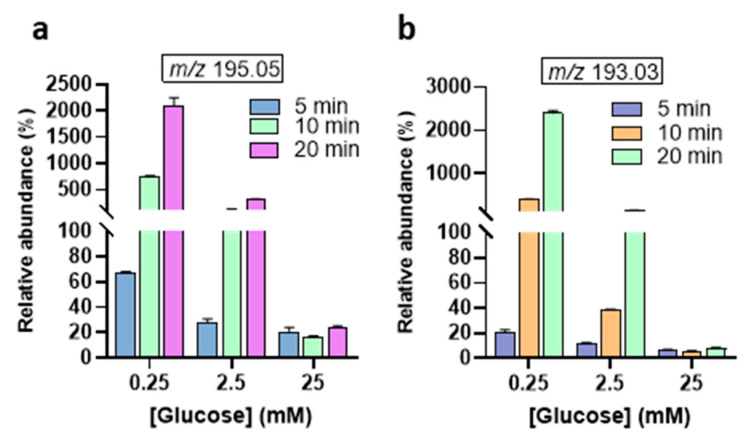
Relative abundances of main GOPs after plasma treatment from 5 to 20 min. (**a**) *D*-gluconic acid, (**b**) *D*-glucuronic acid. The abundance of each oxidation product is expressed as the ratio of its peak area and the peak area of non-oxidized glucose [C_6_H_12_O_6_ − H]^−^ at *m*/*z* 179.05.

**Figure 5:**
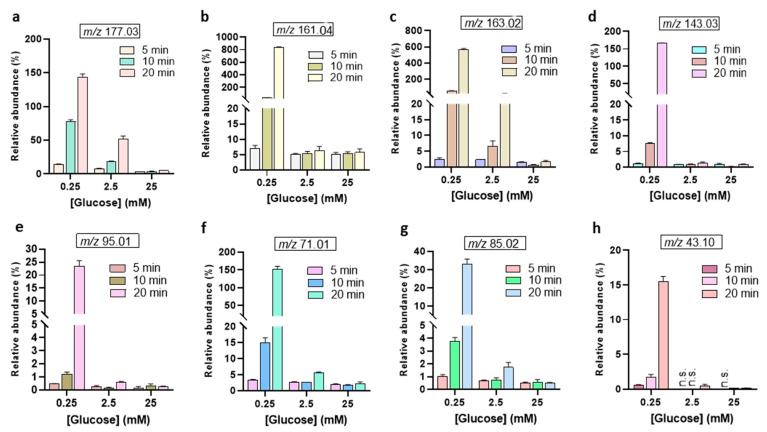
Relative abundances of further glucose oxidation products after plasma treatment from 5 to 20 min. (**a**) 2-keto-*D*-glucose, (**b**) 3DG, (**c**) aldehydo-arabinuronate, (**d**) 3,4DGE, (**e**) furaldehyde, (**f**) methylglyoxal, (**g**) crotonate, (**h**) acetaldehyde. The abundance of each oxidation product is expressed as the ratio of its peak area and the peak area of non-oxidized glucose [C_6_H_12_O_6_ − H]^−^ at *m*/*z* 179.05. n.s. = not seen.

The relative abundances of the MS signal at *m*/*z* 195.04, and *m*/*z* 193.03 were observed to increase with treatment time (Figure 4). Interestingly, the abundance of the 2-keto-*D*-glucose (177.03 *m*/*z*) is lower than the 3DG (161.04 *m*/*z*) (Figure 5). This indicates that the oxidation of the secondary alcohol group of glucose is less relevant compared to the dehydration (see Figure 2B). The calculated abundances for *m*/*z* 177.03, 161.04, and 163.02 showed enhancement at the lower glucose concentration of 0.25 mM. Notably, the *m*/*z* 161.04 signal, corresponding to 3-deoxyglucosone (3DG)— a well-known marker of diabetes [41,42] involved in the formation of advanced glycation end-products (AGEs)—remained consistent at higher glucose concentrations. This suggests that, despite increasing glucose levels, the production of 3DG does not significantly fluctuate, indicating this oxidation product may reach a steady state in higher glucose environments. Indeed, the oxidizing/reactive species generated by the gas plasma are the limiting factor, not glucose, even after 20 min plasma treatment at 25 mM glucose concentrations. The *m*/*z* 143.03, corresponding to the dicarbonyl compound 3,4-dideoxyglucosone-3-ene (3,4DGE), appeared at lower abundance than signals at *m*/*z* 161.04 and 163.02 (Figure 5). Studies have shown that 3,4DGE exhibits toxicity effects on cells in vitro and impairs wound healing [43,44], while also inducing apoptosis in human leukocytes and renal epithelial cells [44]. The abundance of MS signals at *m*/*z* 95.01 and 85.02 was very low for all glucose concentrations (Figure 5). The formation of acetaldehyde (*m*/*z* 43.10) during plasma treatment of glucose is influenced by the pH of the solution [27]. In acidic conditions, which are common in plasma-treated glucose solutions (Supporting Information, Figure S7), the formation of acetaldehyde is less favorable [10].

**Figure 6:**
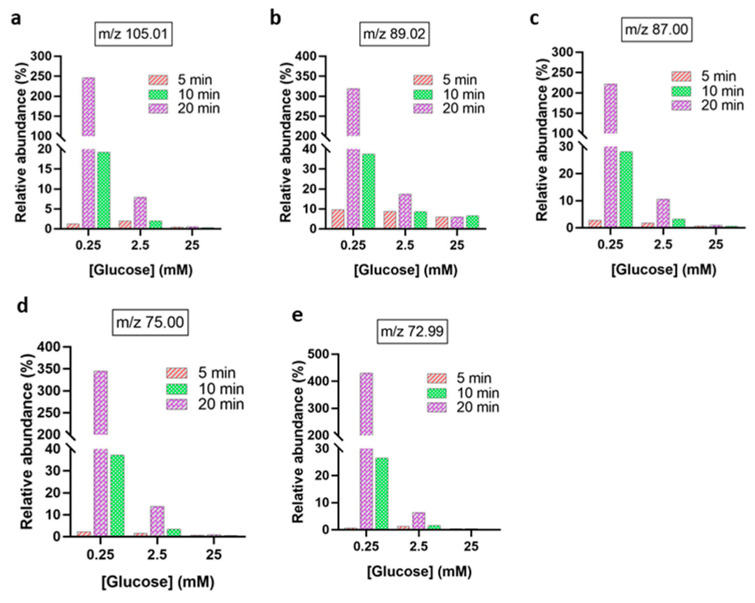
Relative abundances of downstream glucose oxidation products after plasma treatment from 5 to 20 min. (**a**) glycerate [C_3_H_5_O_4_ − H]^−^, (**b**) lactate [C_3_H_5_O_3_ − H]^−^, (**c**) pyruvate [C_3_H_3_O_3_ − H]^−^, (**d**) glycolic acid [C_2_H_4_O_3_ − H]^−^, (**e**) glyoxylate [C_2_HO_3_ − H]^−^. The abundance of each oxidation product is expressed as the ratio of its peak area and the peak area of non-oxidized glucose [C_6_H_12_O_6_ − H]^−^ at *m*/*z* 179.05.

Plasma-induced decomposition of the main GOPs also resulted in a concentration- and time-dependent formation of multiple low-molecular-weight oxidation products (Figure 6), favorably at low initial glucose concentration. The relative abundances of glycerate (*m*/*z* 105.01), lactate (*m*/z 89.02), pyruvate (*m*/*z* 87.00), glycolic acid (*m*/*z* 75.00), and glyoxylate (*m*/*z* 72.99) increased with exposure times (10–20 min). Among these, glyoxylate and glycolic acid exhibited the highest relative abundance. In contrast, higher glucose concentrations (≥2.5 mM) showed a reduced formation of such products, consistent with a glucose concentration-dependent scavenging effect limiting ROS availability for further oxidation. These results demonstrate that plasma-driven glucose oxidation is governed by substrate concentration and exposure duration, leading to distinct patterns of GOPs reflective of stepwise oxidative degradation pathways.

## 4. Conclusions

In conclusion, this study systematically elucidated the oxidative modification pathways of *D*-glucose exposed to plasma-derived reactive oxygen and nitrogen species (RONS) generated by the kINPen MED. High-resolution mass spectrometry (HRMS) and tandem MS/MS enabled precise identification and structural annotation of multiple glucose oxidation products (GOPs), including *D*-gluconic and *D*-glucuronic acids, 2-keto-*D*-glucose, 3-deoxyglucosone (3DG), 3,4-dideoxyglucosone-3-ene (3,4-DGE), furaldehyde, methylglyoxal, glyoxylate, glycerate, crotonate, and acetaldehyde. The results reveal that plasma-induced oxidation proceeds through sequential dehydrogenation, retro-aldol fragmentation, and dehydration reactions, producing low-molecular-weight organic acids and electrophilic carbonyl intermediates. These findings provide mechanistic insight into plasma-driven glucose oxidation chemistry and lay the groundwork for assessing the biological relevance and safety of plasma-tissue or plasma-biomolecule interactions in therapeutic applications where glucose is involved.

## Figures and Tables

**Table 1:** *D*-glucose oxidation modifications after plasma treatments monitored by HRMS and HRMS/MS. ^a^ *m*/*z* 177.03 is primarily assigned to 2-keto-*D*-glucose with minor lactone contribution.

*m*/*z* Values	MS/MS Values	Generic Names [Molecular Ions]
195.04	177.03, 129.01, 99.00, 87.00, 85.00, 75.00, 59.01, and 57.00	*D*-Gluconic acid [C6H12O7 − H]^−^
193.03	165.03, 147.02, 131.03, 113.02, 103.00, 89.02, 85.02, 73.00, 71.01, 59.01, and 45.00	*D*-Glucuronic acid [C6H10O7 − H]^−^
177.03	159.02, 129.01, 117.01, 99.00, 87.00, 71.01, and 59.01	2-Keto-*D*-Glucose (Glucono-1,4-lactone) a [C6H10O6 − H]^−^
163.02	145.01, 103.00, 101.02, 75.00, 73.00, 71.01, 59.01, and 45.00	Aldehydo-arabinuronate [C5H7O6 − H]^−^
161.04	103.00, 99.00, 87.00, 73.00, 71.01, 59.01, and 43.02	3-Deoxyglucosone (3DG) [C6H10O5 − H]^−^
143.03	125.03, 99.00, 97.00, and 71.01	3,4-Dideoxyglucosone-3-ene (3,4DGE) [C6H8O4 − H]^−^
95.01	95.08, 80.00, and 77.00	Furaldehyde [C5H4O2 − H]^−^
85.02	85.10, 69.00, and 53,00	Crotonate [C4H5O2 − H]^−^
71.01	71.01 and 41.00	Methylglyoxal [C3H4O2 − H]^−^
43.10	43.10	Acetaldehyde [C2H4O − H]^−^

**Table 2:** Mass spectra analysis of controls and *D*-glucose solutions before and after plasma treatment in various concentrations diluted with MeOH:H_2_O (0.05% FA) for the final concentration of 125 μM. ^(a)^ MeOH:H_2_O (0.05% FA), ^(b)^ 125 μM glucose in MeOH:H_2_O (0.05% FA). * The results for 20 min plasma treatment and the injected concentration were 125 μM. Values represent relative peak intensities normalized to total ion current (TIC).

*m*/*z* Values	Chemical Formula	Controls and Plasma Treatments *		
		Control ^(a)^ (%)	Control ^(b)^ (%)	Plasma Treatment (%) 0.25, 2.5, 25 mM
61.98	NO_3_^−^	1	~0.5	14, 4, ~0.5
60.99	HCO_3_^−^	0	6	~0.5, 0, 0
59.98	CO_3_^•−^	0	2	6, 1, ~0.5
44.99	HCO_2_^−^	25	25	5, 10, 12

## Data Availability

The original contributions presented in this study are included in the article/Supplementary Material. Further inquiries can be directed to the corresponding authors.

## Supplementary Materials

The following supporting information can be downloaded at: https://www.mdpi.com/article/10.3390/biomedicines13112833/s1, Mass-spectrometry-based glucose peak area after plasma treatment at glucose concentrations of 25 mM, 2.5 mM, and 0.25 mM (Figure S1); Mass spectra of *D*-glucose samples at 0.25 mM (Figures S2 and S3) and after plasma treatment for 5, 10, and 20 min (Figures S4–S6), each diluted to 125 μM in MeOH:H_2_O (1:1, *v*/*v*) with 0.05% FA for direct infusion in negative ionization mode (expanded views of the *m*/*z* range 70–600 Da are shown for each spectrum); pH of water and glucose solutions (0.25–25 mM) after plasma treatment (Figure S7); Mass spectrum (Figure S8) and tandem mass spectra (Figure S9) of 2-keto-*D*-glucose showing characteristic ions at *m*/*z* 177.03, 355.11, and 533.18 and their corresponding fragmentations in negative ionization mode.
