# Supplementary material for: Gas Plasma-Induced Oxidative Transformation of Glucose
Source: Biomedicines. 2025 Nov 20;13(11):2833. doi: 10.3390/biomedicines13112833 (PMC12650470; doi:10.3390/biomedicines13112833)
Supplement: Supplementary file 1 [file biomedicines-13-02833-s001.zip › biomedicines-3949151-supplementary.pdf]

Supplementary Information

Gas plasma-induced oxidative transformation of glucose

Mohsen Ahmadi [1], Kai Masur [1], *Sander Bekeschus [1,2,§] and *Kristian Wende [1,§]

[1] ZIK *plasmatis*, Leibniz Institute for Plasma Science and Technology (INP), Felix Hausdorff-Str. 2, 17489 Greifswald, Germany
[2] Department of Dermatology, Venerology, and Allergology, Rostock University Medical Center, Strempelstr. 13, 18057 Rostock, Germany
* Correspondence: kristian.wende@inp-greifswald.de; sander.bekeschus@inp-greifswald.de
§ equally contributed as last authors.

Content

Pages	Captions
2	**Figure S1.** (a) Mass-spectrometry-based glucose peak area after plasma treatment, (b) percentage of the glucose in solution normalized to untreated samples (100%). Dilution to a nominal concentration of 125 µM with MeOH:H_2O 0.05% FA was performed (1:2, 1:20, 1:200, resp.). Points represent glucose peak area (m/z 179.07 (monomer) + m/z 358.96 (dimer)). The percentage of glucose in solution is defined as the peak area of glucose quantified in each sample relative to the peak area of glucose without plasma treatment at identical condition.
3	**Figure S2**. Mass spectrum of glucose (125 µM) in MeOH:H_2O.
4	**Figure S3**. Mass spectrum of glucose (125 µM) in MeOH:H_2O 0.05% FA.
5	**Figure S4**. Mass spectrum of 0.25 mM glucose solution treated with plasma for 5 min (125 µM injected in MeOH:H_2O 0.05% FA).
6	**Figure S5**. Mass spectrum of 0.25 mM glucose solution treated with plasma for 10 min (125 µM injected in MeOH:H_2O 0.05% FA).
7	**Figure S6**. Mass spectrum of 0.25 mM glucose solution treated with plasma for 20 min (125 µM injected in MeOH:H_2O 0.05% FA).
8	**Figure S7**. pH of water and glucose solutions (0.25–25 mM) after 5 and 20 minutes of plasma treatment. The error bars show range.
9	**Figure S8.** Mass spectrum of 2-keto-d-glucose in the negative mode.
10	**Figure S9.** *D*-glucose MS/MS spectra of the signals at *m/z* 177.05, 355.11, and 533.09 in the negative mode.

Figure S1

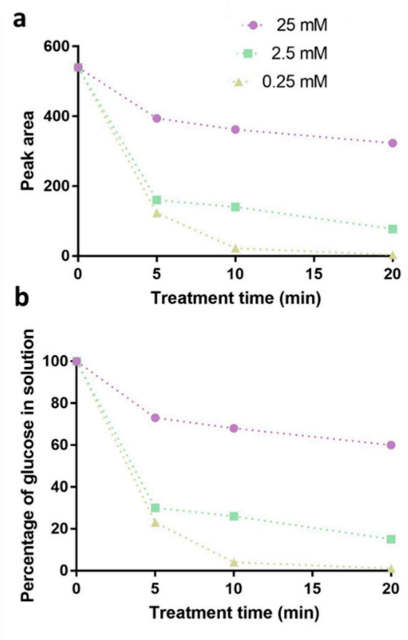
a
25 mM
2.5 mM
0.25 mM
Peak area
600
400
200
0
0
5
10
15
20
Treatment time (min)
b
Percentage of glucose in solution
100
80
60
40
20
0
0
5
10
15
20
Treatment time (min)

Figure S2

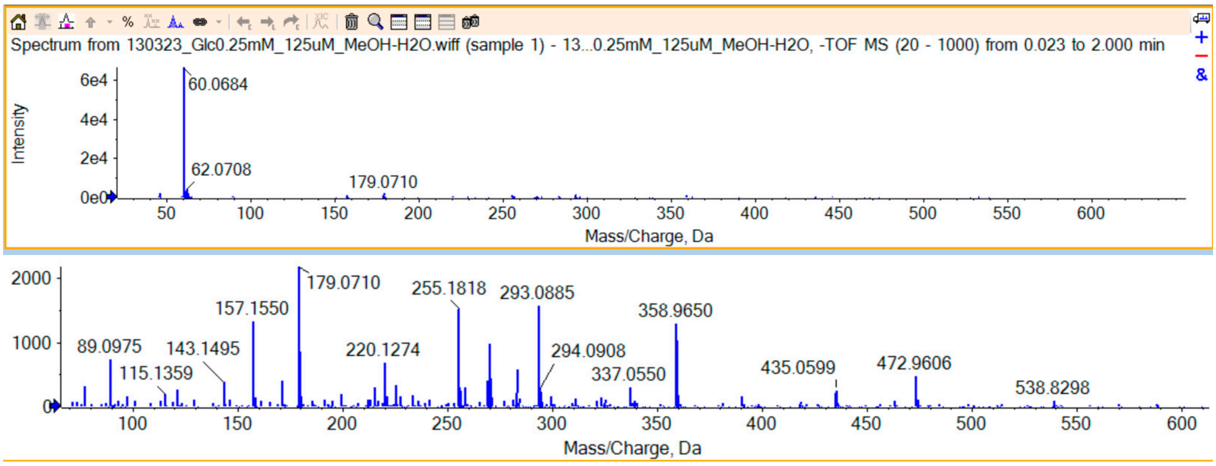

Spectrum from 130323_Glc0.25mM_125uM_MeOH-H2O.wiff (sample 1) - 13...0.25mM_125uM_MeOH-H2O, -TOF MS (20 - 1000) from 0.023 to 2.000 min
Intensity
6e4
4e4
2e4
0e0
60.0684
62.0708
179.0710
Mass/Charge, Da
2000
1000
89.0975
115.1359
143.1495
157.1550
179.0710
220.1274
255.1818
293.0885
294.0908
337.0550
358.9650
435.0599
472.9606
538.8298
Mass/Charge, Da

Figure S3

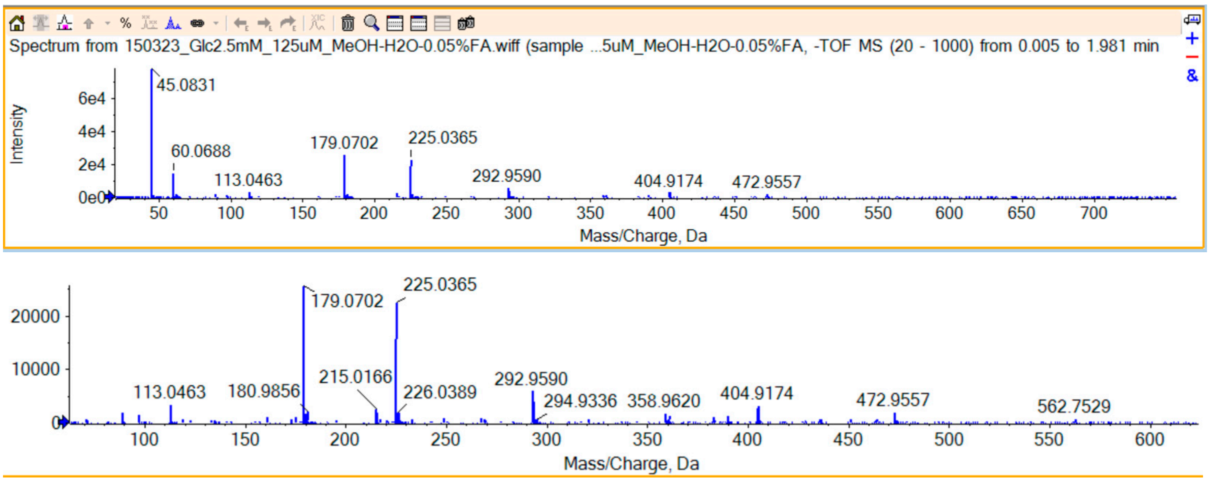
Spectrum from 150323_Glc2.5mM_125uM_MeOH-H2O-0.05%FA.wiff (sample ...5uM_MeOH-H2O-0.05%FA, -TOF MS (20 - 1000) from 0.005 to 1.981 min
Intensity
6e4
4e4
2e4
0e0
45.0831
60.0688
113.0463
179.0702
225.0365
292.9590
404.9174
472.9557
Mass/Charge, Da
20000
10000
0
215.0166
180.9856
226.0389
294.9336
358.9620
562.7529

Figure S4

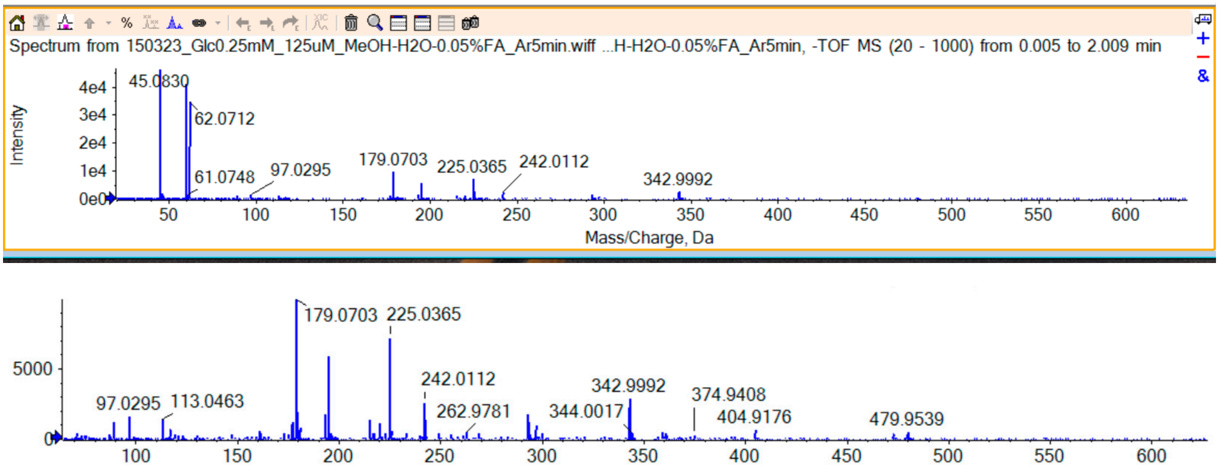
Spectrum from 150323_Glc0.25mM_125uM_MeOH-H2O-0.05%FA_Ar5min.wiff ...H-H2O-0.05%FA_Ar5min, -TOF MS (20 - 1000) from 0.005 to 2.009 min
Intensity
4e4
3e4
2e4
1e4
0e0
45.0830
62.0712
61.0748
97.0295
179.0703
225.0365
242.0112
342.9992
50
100
150
200
250
300
350
400
450
500
550
600
Mass/Charge, Da
5000
0
97.0295
113.0463
179.0703
225.0365
242.0112
262.9781
344.0017
342.9992
374.9408
404.9176
479.9539

Figure S5

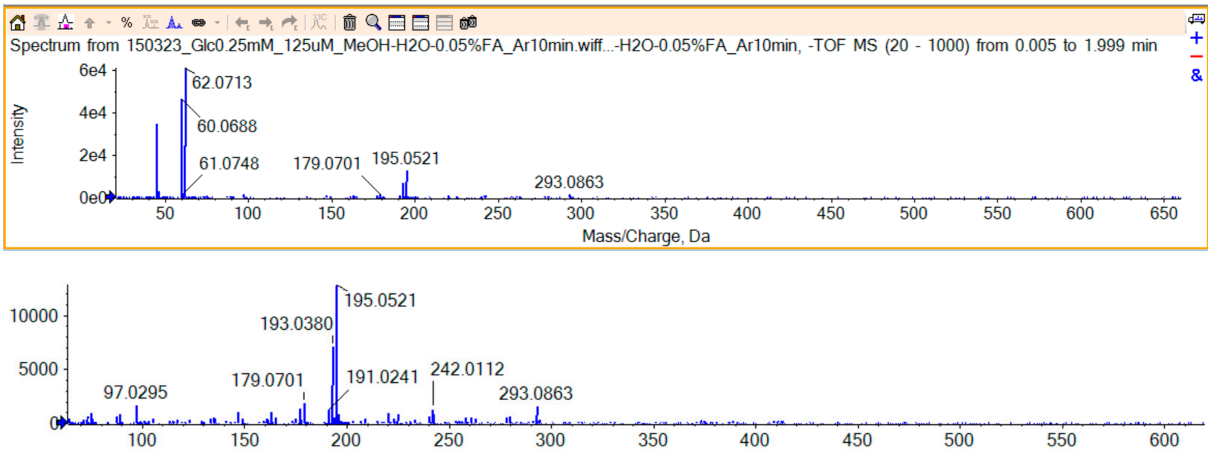
Spectrum from 150323_Glc0.25mM_125uM_MeOH-H2O-0.05%FA_Ar10min.wiff...-H2O-0.05%FA_Ar10min, -TOF MS (20 - 1000) from 0.005 to 1.999 min
Intensity
6e4
4e4
2e4
0e0
62.0713
60.0688
61.0748
179.0701
195.0521
293.0863
50
100
150
200
250
300
350
400
450
500
550
600
650
Mass/Charge, Da
10000
5000
0
195.0521
193.0380
97.0295
179.0701
191.0241
242.0112
293.0863
100
150
200
250
300
350
400
450
500
550
600

Figure S6

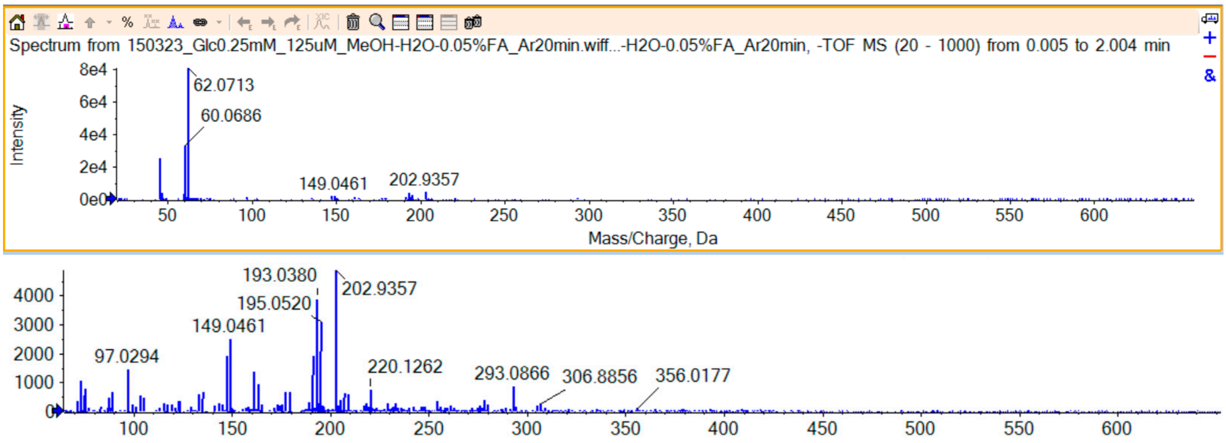
Spectrum from 150323_Glc0.25mM_125uM_MeOH-H2O-0.05%FA_Ar20min.wiff...-H2O-0.05%FA_Ar20min, -TOF MS (20 - 1000) from 0.005 to 2.004 min
Intensity
8e4
6e4
4e4
2e4
0e0
62.0713
60.0686
149.0461
202.9357
50
100
150
200
250
300
350
400
450
500
550
600
Mass/Charge, Da
4000
3000
2000
1000
0
193.0380
195.0520
202.9357
149.0461
97.0294
220.1262
293.0866
306.8856
356.0177
100
150
200
250
300
350
400
450
500
550
600

Figure S7

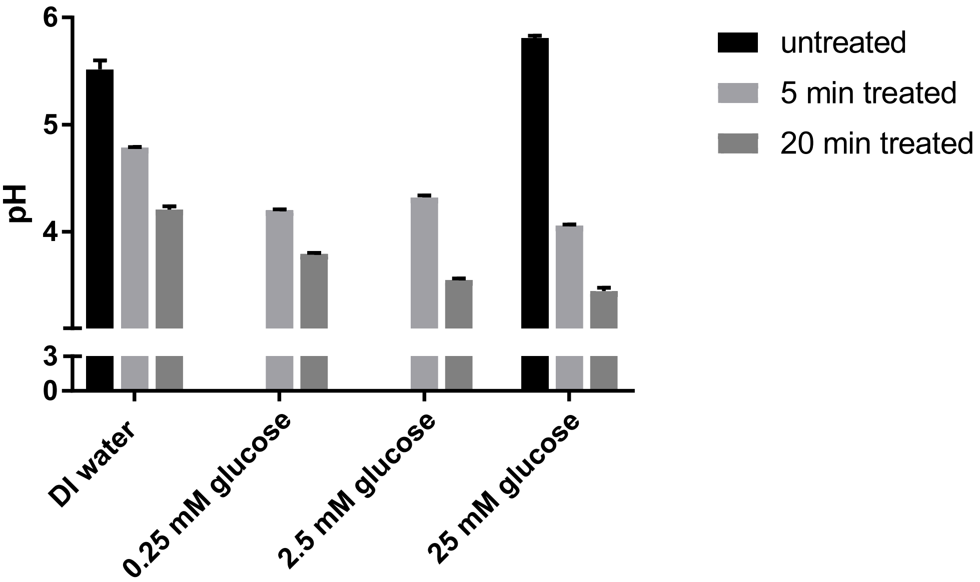
6
5
4
3
0
pH
untreated
5 min treated
20 min treated
DI water
0.25 mM glucose
2.5 mM glucose
25 mM glucose

Figure S8

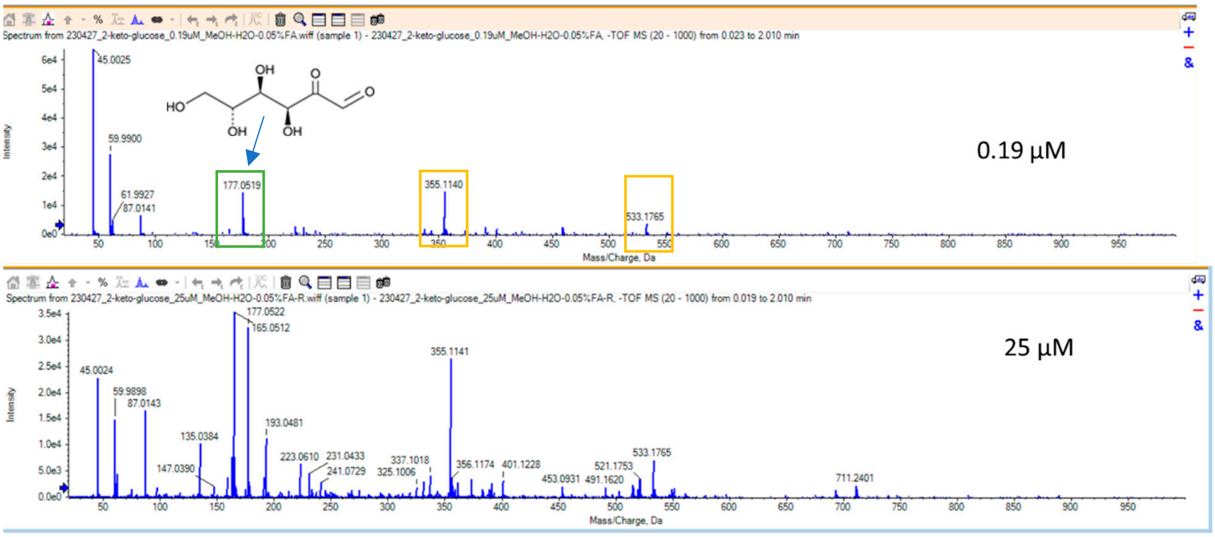

0.19 µM
25 µM
Spectrum from 230427_2-keto-glucose_0.19uM_MeOH-H2O-0.05%FA.wiff (sample 1) - 230427_2-keto-glucose_0.19uM_MeOH-H2O-0.05%FA, -TOF MS (20 - 1000) from 0.023 to 2.010 min
Spectrum from 230427_2-keto-glucose_25uM_MeOH-H2O-0.05%FA-R.wiff (sample 1) - 230427_2-keto-glucose_25uM_MeOH-H2O-0.05%FA-R, -TOF MS (20 - 1000) from 0.019 to 2.010 min
Intensity
Mass/Charge, Da
45.0025
59.9900
61.9927
87.0141
177.0519
355.1140
533.1765
177.0522
165.0512
45.0024
59.9898
87.0143
135.0384
147.0390
193.0481
223.0610
231.0433
241.0729
337.1018
325.1006
355.1141
356.1174
401.1228
453.0931
491.1620
521.1753
533.1765
711.2401
HO
OH
O

Figure S9

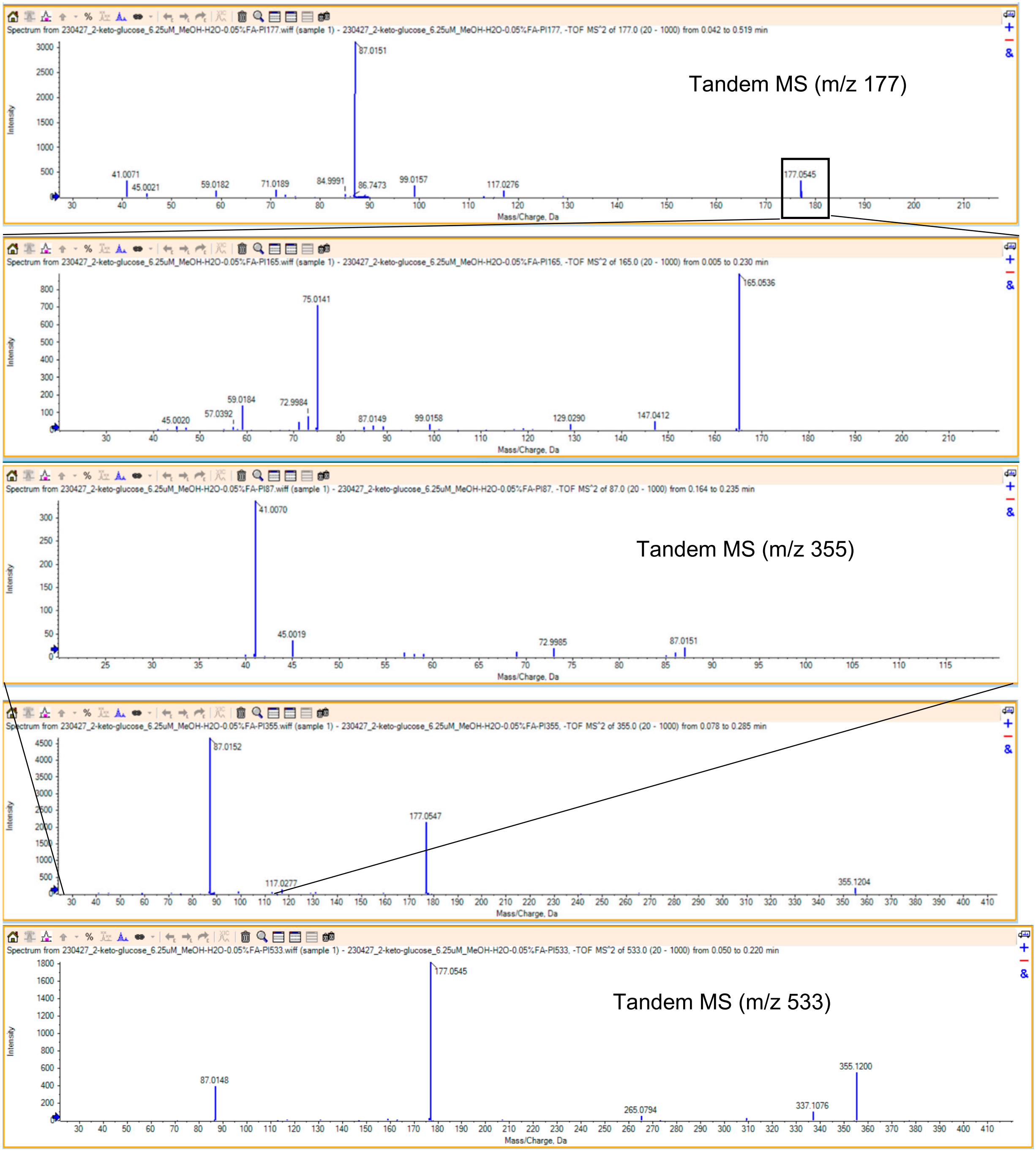
Tandem MS (m/z 177)
87.0151
41.0071
45.0021
59.0182
71.0189
84.9991
86.7473
99.0157
117.0276
177.0545
165.0536
75.0141
59.0184
72.9984
57.0392
45.0020
87.0149
99.0158
129.0290
147.0412
Tandem MS (m/z 355)
41.0070
45.0019
72.9985
87.0151
87.0152
177.0547
117.0277
355.1204
Tandem MS (m/z 533)
177.0545
87.0148
355.1200
337.1076
265.0794
Intensity
Mass/Charge, Da
